# Social support exchange and relationship satisfaction among couples living with HIV: Actor–partner effects of provided and received emotional support

**DOI:** 10.1016/j.ijchp.2026.100665

**Published:** 2026-01-17

**Authors:** Marcin Rzeszutek, Ewa Gruszczyńska, Magdalena Grabowska, Paula Malinowska

**Affiliations:** a Faculty of Psychology, University of Warsaw, Banacha 2D, 02-097 Warsaw, Poland; b Faculty of Psychology, SWPS University, Chodakowska 19/31, 03-815 Warsaw, Poland; c Faculty of Economic Sciences, University of Warsaw, Dluga 44/50, 00-241 Warsaw, Poland; d Department of Health Psychology, Medical University of Warsaw, Litewska 14/16, 00-581, Warsaw, Poland

**Keywords:** HIV/AIDS, Social support, Couples, Relationship satisfaction

## Abstract

This study aimed to examine the associations between both provided and received emotional social support and relationship satisfaction among romantic couples in which at least one partner was HIV positive. In addition, couples’ serostatus concordance and sexual orientation were tested as potential moderators of the effects of social support exchange. A total of 105 couples participated in the study, of whom 46.7% were seroconcordant and 73.3% were same-gender couples. Each partner independently evaluated provided and received emotional social support using the relevant subscales of the Berlin Social Support Scales. Relationship satisfaction was assessed individually using the Relationship Assessment Scale. In the actor–partner interdependence model (APIM), actor and partner effects were comparable for provided support, whereas for received support, the actor effect was slightly stronger than the partner effect. Partners within dyads were empirically classified as indistinguishable. This classification was further supported by the absence of moderating effects of either serostatus concordance or sexual orientation at the dyadic level. The findings suggest that the perceived provision of emotional support functions as a couple-level resource, whereas the perceived receipt of emotional support operates primarily as an individual-level resource for relationship satisfaction. This pattern of support exchange appears consistent across couples, regardless of HIV serostatus or sexual orientation, among our sample of highly functioning people living with HIV who are receiving treatment**.**

A plethora of studies have shown that being in a romantic relationship is crucial for general well-being, health, and longevity (see reviews and meta-analyses, e.g., Buhler et al., 2021; Proulx et al., 2007; Robles et al., 2014). More specifically, it has been observed that people in romantic relationships lead healthier lifestyles and thus have a lower risk of chronic illnesses and mental health (Robles & Kiecolt-Glaser, 2003; Whisman et al., 2018). They also declare greater life satisfaction and meaning than their single counterparts (Be et al., 2013; Heller et al., 2004). The benefits of intimate relationships are also evident among couples struggling with chronic illnesses (Revenson et al., 2005). Traditionally, research in this area has focused only on how patients and their partners adjust to an illness from an individual perspective (Leventhal et al., 1980). However, subsequent studies have found that illness-related distress can significantly impact both partners (Bodenmann, 1995).

Specifically, an illness affects not only the patient’s well-being but also that of their romantic partner, family, and close social environment. Consequently, empirical investigations have begun to focus on how both partners impact each other’s psychological well-being through the exchange of social support within a dyadic framework (Hagedoorn et al., 2008). These mutual processes have been observed in various chronic illnesses, predominantly cancer, myocardial infarction, arthritis, diabetes, and chronic pain Helgeson and Zajdel (2017). To date, little attention has been paid to the dyadic perspective in research on couples living with HIV (e.g., Hou et al., 2025; Martins et al., 2021). In our study, we wanted to examine the relationship between the level of social support provided and received and the perceived relationship satisfaction among couples in which at least one partner was infected with HIV.

Nevertheless, not all couples experience the abovementioned relationship benefits but those who are satisfied with each other (Proulx et al., 2007). Consequently, individuals in “unhappy” relationships typically report poorer physical and lower quality of life than single people (Bühler et al., 2021). Therefore, the fundamental term used to describe intimate relationships is relationship satisfaction. This refers to an individual’s overall assessment of their romantic relationship, including factors such as the extent of their partnership satisfaction, the level of emotional support and intimacy they experience, and the degree of commitment or conflict they encounter (Fincham & Rogge, 2010; Funk & Rogge, 2007). Several studies have shown that there are two major predictors of relationship satisfaction: the age of the partners and the duration of the relationship (Buhler et al., 2021) and the amount of social support received and provided by romantic couples (Kohler et al., 2024). Regarding the former factor, a U-shaped trend was observed as a function of age, as well as a more complex association with relationship duration (the decrease within the first 10 years, followed by growth until 20 years, and then a subsequent decline). When it comes to the exchange of social support, it is still not exactly known to what extent and in what direction partners should provide and receive support from each other to sustain a satisfying intimate relationship (Kohler et al., 2024).

It is well known that the well-being of people living with HIV (PLWH) is positively related to receiving support from close partners (e.g., Abramowitz et al., 2009; Earnshaw et al., 2013; Kim et al., 2015). Nevertheless, PLWH sometimes have concerns about receiving support, as they wish to spare their partners the emotional distress connected with providing support due to HIV/AIDS stigma (Takada et al., 2014). This seems to support the “mixed blessing of receiving support” hypothesis (Gleason et al., 2008). In contrast, the importance of providing social support is much worse in the HIV/AIDS context, despite increasing research suggesting that it may promote well-being more effectively than receiving support (Kroemeke & Gruszczyńska, 2016; Morelli et al., 2015). In line with this trend, Rzeszutek and Gruszczyńska (2018) showed in a daily diary study that providing support has a better positive effect on the affective well-being of PLWH compared to receiving such support. However, if not reciprocated in the longer term, providing support may have a detrimental effect on the provider’s well-being (Bolger et al., 2000; Kroemeke et al., 2019). Therefore, in our study, we focused on the phenomenon of social support exchange between couples living with HIV (Wang & Gruenewald, 2019). Until now, the vast majority of research has concentrated on support exchange in the context of receiving mutual receiving in romantic relationships (see reviews and meta-analyses, e.g., Buhler et al., 2021; Weitkamp et al., 2021). Much less attention has been devoted to the positive effects of providing support in such relationships (Rzeszutek & Gruszczyńska, 2018; Kohler et al., 2024) and even less to mutual relationship satisfaction in couples (Wang & Gruenewald, 2019).

In couples living with HIV, there is one additional factor that may complicate the abovementioned associations, namely the ongoing and pervasive HIV/AIDS stigma and the structure of such couples. Specifically, in a prospective study, Huang et al. (2018) observed that the ability to shift from identifying as a “separate self” to identifying as a “couple” significantly reduced the intensity of the HIV/AIDS stigma experienced by couples living with HIV. However, the stigma experienced by serodiscordant couples (where one partner is HIV positive and the other is HIV negative) may differ from that experienced by seroconcordant couples (where both partners are HIV positive; Muessig & Cohen, 2014). For example, Siegel et al. (2018) found that the spouses of PLWH who are seronegative may experience courtesy stigma, which includes public avoidance, due to society’s tendency to treat people in serodiscordant couples as “one.” In contrast, seroconcordant couples may experience stigmatization differently due to their shared HIV-positive status and the related societal reactions to it (Muessig & Cohen, 2014). However, these previous studies have several methodological shortcomings, including the inappropriate use of statistical methods for the dyadic model, so the exact mechanisms through which partners support each other in the context of HIV/AIDS are still not fully understood (Hou et al., 2023, 2025).

## Current study

To address these limitations, we employed the actor–partner interdependence model (APIM), a well-established framework for examining interpersonal exchange in dyads (Kenny et al., 2006, 2020). This model captures interdependence within close relationships, wherein the emotions, cognitions, or behaviors of one individual influence those of their partner. Dyads may be indistinguishable when members cannot be meaningfully differentiated on a given variable or distinguishable when such differentiation is possible (Kashy et al., 2008).

In the context of HIV/AIDS, both configurations may occur in terms of HIV status. Consequently, we were particularly interested in whether homogeneity in HIV status functions as a resource or as an additional burden within couples. Similarly, individuals may form either homogenous or heterogeneous couples with respect to sexual orientation. Global surveillance data indicate that men who have sex with men constitute a disproportionately large segment of PLWH, whereas heterosexual women comprise a comparatively small proportion in many high-income countries (UNAIDS, 2023). Given these differing minority statuses and their associated psychosocial burdens (Gruszczyńska & Rzeszutek, 2023), partnership composition may influence relationship satisfaction.

Hence, our study focuses on the social support within romantic relationships which is a key predictor of relationship satisfaction (Kohler et al., 2024). Although earlier studies focused mainly on receiving support, growing evidence suggests that providing support may be equally or even more beneficial for well-being (Morelli et al., 2015; Kroemeke & Gruszczyńska, 2016). In the context of HIV/AIDS, providing support has been shown to have stronger positive effects on affective well-being than receiving support (Rzeszutek & Gruszczyńska, 2018), although these benefits may diminish when support is not reciprocated over time (Bolger et al., 2000; Kroemeke et al., 2019). Despite these findings, dyadic effects of support provision on both partners’ relationship satisfaction remain understudied (Bühler et al., 2021; Weitkamp et al., 2021). Building on this literature, the present study hypothesizes that higher levels of emotional support provided by an individual will be positively associated with both their own and their partner’s relationship satisfaction, after accounting for dyadic interdependence (hypothesis 1; H1).

As already mentioned, receiving emotional support from a partner is a well-established predictor of well-being and relationship satisfaction, also among PLWH (Abramowitz et al., 2009; Earnshaw et al., 2013; Kim et al., 2015). At the same time, support receipt in this context may be psychologically complex, as some individuals hesitate to accept support to protect their partners from emotional strain or stigma-related stress (Takada et al., 2014). These findings highlight the need for a dyadic perspective, as receiving support may affect not only the recipient but also the partner. Accordingly, drawing on dyadic models of close relationships, our second hypothesis (H2) is that greater emotional support received by an individual will be positively associated with both their own and their partner’s relationship satisfaction.

Finally, the associations between emotional support and relationship satisfaction may further depend on the dyadic context of couples living with HIV. HIV serostatus composition has been shown to shape experiences of HIV/AIDS stigma, with serodiscordant and seroconcordant couples facing distinct challenges (Muessig & Cohen, 2014; Siegel et al., 2018). Moreover, adopting a shared couple identity may buffer stigma-related stress (Huang et al., 2018). Similarly, sexual composition (gay/lesbian, heterosexual, or bisexual) is linked to differing minority stressors that may influence relationship processes (Gruszczyńska & Rzeszutek, 2023). Therefore, the current study also posits that couples’ HIV serostatus and sexual composition will moderate the associations between both provided and received emotional support and relationship satisfaction (hypothesis 3; H3).

Summing up, our goal was to examine potential interdependence in the exchange of social support within couples in which at least one member of the dyad (the identified patient) has an HIV diagnosis, with individual relationship satisfaction specified as the outcome variable. In addition, we aimed to determine whether seroconcordant versus serodiscordant HIV status and sexual orientation moderate this exchange at the dyadic level.

## Method

### Participants and procedure

Recruitment was conducted at the outpatient clinic of an infectious disease hospital specializing in HIV/AIDS diagnosis and treatment. Participants were enrolled during their checkup visits related to their antiretroviral treatment between September 2024 and June 2025. Only individuals with a medically confirmed HIV-positive diagnosis were approached. Additional inclusion criteria required participants to be at least 18 years old, to be receiving antiretroviral treatment at the clinic where the study was conducted, and to be currently involved in a romantic relationship. Individuals with HIV-related cognitive disorders or active psychoactive substance abuse, as evaluated by medical staff, were excluded. After providing written informed consent, the participants completed a set of paper-and-pencil questionnaires.

The partners of the patients were recruited using two procedures. First, when partners accompanied patients to their outpatient visits, they were approached directly and, after providing informed consent, were invited to complete the survey on site. Second, when partners were not present, the patients provided their contact information. A research assistant then contacted the partners, explained the study, and obtained their informed consent. Subsequently, a personalized link to the online Qualtrics questionnaire was sent, with a seven-day window for completion. The study protocol received approval from the institutional ethics committee.

The final sample comprised 105 couples (210 individuals). The individual characteristics of the participants are presented in Table 1. As shown, the identified patients (i.e., the HIV-positive partners in each couple) were predominantly men (82 %), 70 % were gay/lesbian, and their mean age was 39 years (*SD* = 9.8). Among these patients, 61 % held a higher education degree, 80 % were stably employed, and 63 % reported at least a good financial situation. On average, they had been living with HIV for 12.0 years (*SD* = 8.8). Additionally, 12.6 % had been diagnosed with AIDS, and 96.1 % had an undetectable viral load, indicating effective treatment.

**Table 1:** Individual (*n* = 210) characteristics of the studied sample.

Variable	Patients n ( %)	Partners n ( %)
Gender		
Man	86 (81.9 %)	95 (90.5 %)
Woman	19 (18.1 %)	8 (7.6 %)
Not disclosed	-	2 (1.9 %)
Age (*M* ± SD)	38.99 (9.85)	39.63 (9.84)
Education		
Primary and vocational	6 (5.7 %)	10 (9.5 %)
Secondary	35 (33.3 %)	27 (25.7 %)
University degree	64 (61.0 %)	68 (64.8 %)
Employment		
Stable employment	86 (81.9 %)	89 (85.6 %)
Unemployment	12 (11.4 %)	10 (9.6 %)
Retirement or ill health pension	7 (6.7 %)	5 (4.8 %)
Financial Situation		
Good or very good	67 (63.8 %)	70 (66.7 %)
Fair	32 (30.5 %)	28 (26.7 %)
Bad or very bad	6 (5.7 %)	7 (6.7 %)
Sexual Orientation		
Heterosexual	25 (23.8 %)	23 (21.9 %)
Gay/lesbian	73 (69.5 %)	74 (70.5 %)
Bisexual	7 (6.7 %)	8 (7.6 %)
AIDS diagnosis	13 (12.6 %)	7 (13.7 %)
Detectable viral load	4 (3.9 %)	1 (2.04 %)
HIV infection duration in years (*M* ± SD)	11.97 (8.77)	11.50 (7.35)

*Notes: M* mean*, SD* standard deviation.

Their partners were 46.7 % HIV positive and were predominantly men, with a mean age of 39.6 years (*SD* = 9.8). Similar to the patients, 64.8 % of partners held a higher education degree, 85.6 % had stable employment, and 66.7 % reported at least a good financial situation. As shown in Table 2, HIV-positive and HIV-negative partners differed significantly only in sexual orientation, with a greater proportion of HIV-negative partners identifying as gay/lesbian.

**Table 2:** Sociodemographic characteristics of HIV-Positive (*n* = 49) and HIV-Negative (*n* = 56) partners of the identified patients (always HIV Positive, *n* = 105).

Variable	Partners HIV+ *n* ( %)	Partners HIV- n ( %)	Statistical difference
Gender			
Man	41 (83.7 %)	54 (96.4 %)	*χ*2(2) = 5.84, *p* = .054
Woman	7 (14.3 %)	1 (1.8 %)	
Not disclosed	1 (2.0 %)	1 (1.8 %)	
Age (*M* ± SD)	41.58 (9.56)	37.96 (9.86)	*t*(102) = −1.89, *p* = .061
Education			
Primary and vocational	6 (12.2 %)	4 (7.2 %)	*χ*2(3) = 3.00, *p* = .392
Secondary	15 (30.6 %)	12 (21.4 %)	
University degree	28 (57.2 %)	40 (71.4 %)	
Employment			
Stable employment	38 (79.2 %)	51 (91.1 %)	*χ*2(2) = 3.10, *p* = .212
Unemployment	7 (14.6 %)	3 (5.4 %)	
Retirement or ill health pension	3 (6.3 %)	2 (3.6 %)	
Financial Situation			
Good or very good	32 (65.3 %)	38 (67.9 %)	*χ*2(4) = 0.57, *p* = .966
Fair	13 (26.5 %)	15 (26.8 %)	
Bad or very bad	4 (8.2 %)	3 (5.4 %)	
Sexual Orientation			
Heterosexual	12 (21.5 %)	11 (19.6 %)	*χ*2(2) = 6.76, *p* = .034
Gay/lesbian	30 (61.2 %)	44 (78.6 %)	
Bisexual	7 (14.3 %)	1 (1.8 %)	

*Notes: M* mean*, SD* standard deviation; *χ2* Pearson’s Chi-squared test of independence, *t* Student’s *t-*test for independent samples.

At the dyadic level (see Table 3), 56 couples were serodiscordant, and 49 were seroconcordant. Regarding sexual orientation, 25.2 % of the participants were in heterosexual relationships, whereas 74.8 % were in same-sex relationships. The average relationship length was 7.88 years (SD = 7.08).

**Table 3:** Characteristics of Couples (*N* = 105) at the Dyadic Level.

Variable	n ( %)
Couple’s Serostatus	
Seroconcordant	49 (46.7 %)
Serodiscordant	56 (53.3 %)
Couple’s gender composition	
Same-sex	77 (74.8 %)
Different-sex	26 (25.2 %)
Couple’s orientation composition	
Gay/lesbian	70 (67.3 %)
Heterosexual	22 (21.2 %)
Bisexual	12 (11.5 %)
Relationship length in years (*M* ± SD)	7.88 (7.08)

*Notes: M* mean*, SD* standard deviation; couple’s orientation was coded as bisexual if at least one partner declared being bisexual.

## Measures

### Relationship satisfaction

The Relationship Assessment Scale (RAS, Hendrick et al., 1998) was used in the Polish adaptation by Adamczyk et al. (2022) to assess relationship satisfaction. The scale comprises seven items (e.g., “To what extent does your partner fulfill your needs?”) rated on a 5-point Likert scale ranging from 1 (not at all) to 5 (to a very great extent). Higher scores indicate greater relationship satisfaction. RAS has been widely used internationally to assess relationship satisfaction across diverse samples (Graham et al., 2011). Confirmatory factor analysis in the Polish sample (*N* = 733) supported a clear one-factor structure, and the scale demonstrated good internal consistency (Adamczyk et al., 2022). Additionally, measurement invariance was established across countries (Poland, Hungary, the United States), gender, and relationship type (formal vs. informal). Convergent, divergent, and concurrent validity were also supported through theoretically consistent associations with related constructs, including a strong positive correlation with intentions to continue the relationship (Adamczyk et al., 2022). In our study, consistent with the previous finding, Cronbach’s alpha was high, with 0.87 for the identified patients and 0.90 for their partners.

### Emotional social support: received and provided

Berlin Social Support Scales (BSSS, Schulz & Schwarzer, 2003) in the Polish adaptation by Łuszczyńska et al. (2002) were used to assess self-reported received and provided emotional support by each individual within the dyad. The received support subscale includes nine items (e.g., “My partner showed me that they love and accept me”). The provided support subscale also contains nine items and mirrors the received support items, differing only in the direction of the support exchange (e.g., “I showed this person how much I cherish and accept them”). Responses were given on a 4-point Likert-type scale ranging from 1 (definitely untrue) to 4 (definitely true), with higher scores indicating greater self-evaluated support received and provided. The BSSS has been used in previous dyadic research (Knoll et al., 2006), including studies on coping with chronic illness (Luszczynska et al., 2007; Schulz & Schwarzer, 2004), demonstrating medium to satisfactory internal consistency and good test–retest reliability. In this study, Cronbach’s alpha values for received and provided support were 0.83 and 0.81 for the identified patients and 0.85 and 0.79 for partners, respectively.

### Sociodemographic and clinical variables

After providing informed consent, all the participants completed a sociodemographic survey. They reported their year of birth, gender, education, employment, financial situation, and sexual orientation. Individuals living with HIV (the identified patients and HIV-positive partners) also provided clinical information, including year of diagnosis, AIDS history, and most recent viral load (detectable vs. undetectable).

### Dyadic-level variables

At the dyadic level, three variables were assessed: HIV status concordance versus discordance, sexual orientation of the couple, and relationship length. HIV status concordance was coded to distinguish seroconcordant couples (both partners HIV positive) from serodiscordant couples (only one partner HIV positive, identified as the patient). Sexual orientation was coded to differentiate same-sex couples from heterosexual and bisexual couples. Couples were classified as bisexual if at least one partner self-identified as bisexual.

### Data analysis

Preliminary analyses included missing data analysis and descriptive statistics, with testing for possible sociodemographic covariates of the studied variables. This part of the analysis was done with STATA18.

For hypothesis testing, we applied APIM to examine both intrapersonal (actor) and interpersonal (partner) associations between social support exchange and relationship satisfaction (Kenny et al., 2006). Separate models were estimated for received and provided support. For each model, an individual’s predictor score was specified to predict both their own outcome (actor effect) and their partner’s outcome (partner effect) while accounting for dyadic nonindependence by correlating the residuals of corresponding indicators across partners.

To evaluate the relative magnitude of actor and partner effects, the *k* parameter was estimated as the ratio of the partner effect to the actor effect. The *k* parameter provides a summary index of dyadic interdependence and indicates whether influence within the dyad is primarily intrapersonal (actor driven), interpersonal (partner driven), or reciprocal. Values of *k* equal to 0 indicate the absence of partner effects, whereas values of *k* equal to 1 reflect the equivalence of actor and partner effects. Values between 0 and 1 suggest weaker partner effects relative to actor effects, values greater than 1 indicate stronger partner influence, and negative values signal effects operating in opposite directions.

Prior to estimating the APIM, we evaluated whether dyad members should be treated as distinguishable or indistinguishable (Kenny et al., 2006), as both options were theoretically plausible in our study based on the HIV status of the partner of the identified patient (who was always HIV positive). Rather than imposing this distinction a priori, we treated distinguishability as an empirical question. Following established recommendations, we compared a model in which all structural paths, variances, and covariances were constrained to equality across dyad members with an unconstrained model. A significant decrease in model fit indicates that dyad members are not exchangeable and should be treated as distinguishable (Kenny et al., 2006). HIV status concordance versus discordance and the couple’s sexual orientation were then introduced as potential dyadic-level moderators of actor and partner effects. These moderators also served as an additional sensitivity check regarding whether partners within couples functioned as distinguishable or indistinguishable members.

Model fit was assessed using the chi-square test, the comparative fit index (CFI), the Tucker–Lewis index (TLI), the root mean square error of approximation (RMSEA) with a 90 % confidence interval (CI), and the standardized root mean square residual (SRMR). All models were conducted in R using the lavaan package. Parameter estimates were interpreted using standardized coefficients and associated CIs. All analyses were performed at the dyadic level with couple ID specified to structure pairwise data.

## Results

### Descriptive statistics and missing data analysis

Table 4 presents descriptive statistics for the provided and received emotional social support for the identified patients and their partners. As shown, all variables exhibited negative skewness and positive kurtosis, indicating a tendency to cluster scores at the higher end of the response scales and leptokurtic distributions. Given the characteristics of the sample, such distributions are not unexpected, as individuals in stable romantic relationships typically report high levels of social support and satisfaction with relationships (e.g., Hsieh & Hawkley, 2018). Nonetheless, these values remain acceptable for the analytical model used in this study (Hair et al., 2022).

**Table 4:** Individual-level descriptive statistics for relationship satisfaction, provided emotional support, and received emotional support.

Variable		M	SD	Range	Skewness	Kurtosis
Relationship satisfaction	Patients	28.88	4.50	12–35	−1.28	2.14
	Partners	28.92	4.76	12–35	−1.29	1.48
Provided Emotional Support	Patients	32.56	3.71	22–36	−0.99	0.12
	Partners	32.07	3.93	19–36	−1.11	0.81
Received Emotional Support	Patients	31.59	4.60	19–36	−1.12	0.36
	Partners	31.61	4.71	15–36	−1.41	1.83

*Notes: M* mean*, SD* standard deviation.

In the regression analyses, with the study variables treated as outcomes and individual sociodemographic characteristics entered as predictors, no significant associations were found for the identified patients. For partners, the only significant relationship was noted for self-evaluation of financial situation; those who rated their financial situation as bad or very bad reported significantly lower relationship satisfaction and levels of received support.

Finally, there were only minimal missing data, with a maximum of 8 missing cases on the partner-reported provided support variable, corresponding to approximately 7.6 % of the relevant dataset (*n* = 105). Missing data were handled using full information maximum likelihood (FIML), which yields unbiased parameter estimates under missing-at-random assumptions.

### Hypothesis testing

#### Provided emotional social support

For the provided social support exchange, partners in the dyads could be treated as indistinguishable (*Δχ*²(6) = 6.75, *p* = .35). The final model demonstrated a good fit, *χ*²(6) = 6.75, *p* = .35, *CFI* = 0.994, *TLI* = 0.994, *RMSEA* = 0.04, 90 % CI [.00, 0.14], *SRMR* = 0.07. The results for APIM with indistinguishable partners are presented in Table 5.

**Table 5:** Actor and partner effects on relationship satisfaction and dyadic-level moderation by HIV status concordance and sexual orientation.

Predictor	B	SE	95 % CI	β	p
*Provided Emotional Support*					
*Individual-level effects*					
Actor effect	0.50	0.09	[0.33, 0.68]	.41	< 0.001
Partner effect	0.42	0.08	[0.28, 0.58]	.34	< 0.001
Residual variance	13.19				
R²	.39				
Intraclass correlation	.29		[0.04, 0.52]		.02
k parameter	0.83		[0.50, 1.38]		
*Dyadic-level moderating effects*					
HIV status					
HIV status x Actor effect	0.10	0.11	[−0.13,0.31]	.35	.38
HIV status x Partner effect	−0.13	0.12	[−0.35,0.10]	−.45	.25
Sexual orientation					
Sexual orientation (heterosexual) x Actor effect	0.05	0.14	[−0.24,0.30]	.16	.70
Sexual orientation (heterosexual) x Partner effect	−0.06	0.14	[−0.31,0.22]	−.18	.65
Sexual orientation (bisexual) x Actor effect	−0.17	0.24	[−0.39,0.33]	−.33	.49
Sexual orientation (bisexual) x Partner effect	0.15	0.26	[−0.37,0.43]	.28	.57
*Received Emotional Support*					
*Individual-level effects*					
Actor effect	0.54	0.06	[0.42,0.66]	.54	< 0.001
Partner effect	0.31	0.07	[0.18,0.45]	.31	< 0.001
Residual variance	10.79				
R²	0.48				
Intraclass correlation	0.19		[−0.07,0.41]		.36
k parameter	0.58		[0.34,0.90]		
*Dyadic-level moderating effects*					
HIV status					
HIV status x Actor effect	−0.05	0.09	[−0.27,0.10]	−.17	.61
HIV status x Partner effect	0.03	0.09	[−0.12,0.26]	.11	.73
Sexual orientation					
Sexual orientation (heterosexual) x Actor effect	0.04	0.09	[−0.13,0.22]	.11	.69
Sexual orientation (heterosexual) x Partner effect	−0.04	0.09	[−0.23,0.13]	−.12	.67
Sexual orientation (bisexual) x Actor effect	−0.09	0.17	[−0.31,0.19]	−.18	.60
Sexual orientation (bisexual) x Partner effect	0.07	0.17	[−0.21,0.31]	.15	.67

*Notes. N* = 105 dyads, *n* = 210 individuals. Dyads were treated as indistinguishable.

**Fig. 1:**
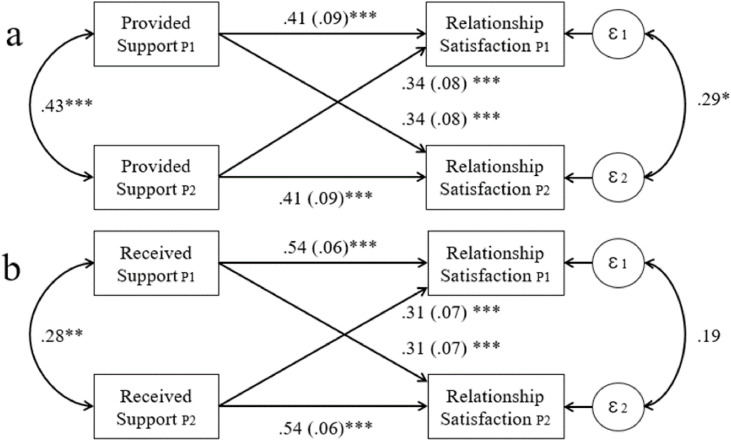
Actor–partner independence models for indistinguishable dyads testing a relationship between emotional support exchange and relationship satisfaction.

*Notes*: * *p* < .05, **p < .0.01, ***p < .001, Ɛ – standard errors of estimates. Horizontal arrows represent actor effects, crossed arrows represent partner effects, and curved (bent) arrows represent correlation.

The model explained 39.2 % of the variance in relationship satisfaction. The partial intraclass correlation for relationship satisfaction, controlling for the predictor variables, was 0.29 (*p* = .016, 95 % CI [0.04, 0.52]), indicating that partners’ scores on relationship satisfaction tended to covary within dyads. The actor effect was 0.50 (*p* < .001, 95 % CI [0.33, 0.68]), with a standardized value of 0.41. The partner effect was also significant and equaled 0.42 (*p* < .001, 95 % CI [0.28, 0.57]), with a standardized value of 0.34. This model is presented in Fig. 1a.

Given the significance of the actor effect, the *k* parameter was interpreted. The estimated *k* was 0.83. Bootstrapped CI indicated that the couple pattern (*k* = 1) was plausible, as the 95 % percentile interval ranged from 0.49 to 1.38. Thus, Hypothesis 1 was confirmed, suggesting a couple-oriented pattern (Flitzpatrick et al., 2016).

#### Received emotional social support

For the received support exchange, the indistinguishable character of partners within dyads was obtained (*Δχ*²(6) = 4.98, *p* = .55). The model was very well fitted to the data, *χ*²(6) = 4.98, *p* = .55, *CFI* = 1.0, *TLI* = 1.0, *RMSEA* = 0.00, 90 % CI [.00, 0.12], *SRMR* = 0.04. As can be seen in Table 5, the model explained 47.6 % of the variance in relationship satisfaction. The partial intraclass correlation for relationship satisfaction was 0.19 and was not statistically significant (*p* = .36, 95 % CI [−.07, 0.41]), indicating a limited within-dyad covariation of the outcome in this model. The actor effect was 0.54 (*p* < .001, 95 % CI [0.42, 0.66]), with a standardized estimate of 0.54. The partner effect was 0.31 (*p* < .001, 95 % CI [0.18, 0.45]), with a standardized estimate of 0.31. This model is presented in Fig. 1b

The *k* parameter was 0.58. Bootstrap estimates indicated that the dyadic pattern fell between the actor-only model (*k* = 0) and the couple model (*k* = 1), as the 95 % percentile CI ranged from 0.34 to 0.90. This result suggests that although the partner effect was significant and operated in the same direction as the actor effect, it was likely weaker in magnitude. Thus, Hypothesis 2 was confirmed, additionally suggesting an actor-oriented pattern (Flitzpatrick et al., 2016).

Additionally, although the standardized coefficients for provided emotional support (*β* = 0.41) and received emotional support (*β* = 0.54) differ somewhat, comparisons should be based on the unstandardized estimates (Kenny et al., 2006). The small difference between these coefficients (0.04; *B* = 0.50, *SE* = 0.09 vs. *B* = 0.54, *SE* = 0.05) and the substantial overlap in their confidence intervals suggest that the effects of provided and received emotional support on relationship satisfaction are of a similar magnitude.

#### HIV status and sexual orientation as dyadic-level moderators

Moderation was examined by including interaction terms between each dyadic moderator (HIV status concordance and sexual orientation) and the indistinguishable actor and partner effects. Because actor and partner paths were constrained to be equal across dyad members, the moderation test assessed whether the shared actor and shared partner effects varied as a function of the moderator. The relevant interaction terms were added to each model (moderator × actor predictor and moderator × partner predictor). A significant interaction would indicate that the magnitude of the common actor or partner effect differs across the levels of the moderator. For both moderators, all interaction terms were nonsignificant (see Table 5). Thus, neither HIV status concordance nor sexual orientation moderated the actor or partner effects in the provided or received emotional support models obtained earlier. Hypothesis 3 was not supported by the data.

Estimates were obtained using FIML to handle missing data. Standardized estimates (β) represent standardized path coefficients. Categorical dyadic-level moderators were coded as follows: HIV status concordance coded as 1 with 0 for HIV status discordance; sexual orientation coded as 0 for gay/lesbian couples, 1 for heterosexual couples, and 2 for bisexual couples.

## Discussion

The results were consistent with our first two hypotheses, indicating that both providing and receiving support had significant effects on the relationship satisfaction of the identified patients and their romantic partners. However, although providing support showed comparable associations with relationship satisfaction at both the actor and partner effects, receiving support was more strongly associated with the actor than with the partner effect. The findings indicate that the experience of receiving support contributes more strongly to individuals’ own relationship satisfaction than to their partners’ satisfaction. In contrast, the effects of providing support appear relatively balanced across both members of the dyad. This pattern reflects an asymmetry in support processes within couples: the intrapersonal (actor) effect of receiving support is substantial, whereas its interpersonal (partner) effect is comparatively smaller. Such a configuration suggests that the perceived provision of emotional support operates as a relational resource within the couple, whereas the perceived receipt of emotional support functions primarily as a personal resource within the dyadic system. Still, the explained variance in relationship satisfaction was higher for received support than for provided support. Moreover, the dyads in this study were empirically indistinguishable, and additional analyses confirmed that neither serostatus concordance nor sexual orientation composition altered these associations, providing no support for Hypothesis 3.

Although our APIM results did not directly assess the balance of support exchange, they can be meaningfully situated within the broader framework of social exchange theories. Our findings may be discussed in light of three classic theories of social exchange. First, equity theory proposes that relative symmetry in receiving and providing support is central for maintaining relationship functioning and deriving emotional benefits from partnerships (Adams, 1966).

In our study, the similar actor and partner effects of providing emotional support on relationship satisfaction may reflect a shared understanding that intimate relationships involve a mutual obligation to offer support, which, in turn, promotes the well-being of both partners in the short and long term (Gleason et al., 2008; Rzeszutek & Gruszczyńska, 2018). This interpretation is consistent with the distributions of the measured variables, as both emotional support and relationship satisfaction showed predominantly high values, indicating that most participants reported substantial provided and received support and satisfaction in their current relationships. However, there is still a matter of debate regarding the extent to which such behavior reflects a true tendency to care for partners, or maybe it derives from external moral norms to reciprocate in all social exchanges (see the norm of reciprocity theory; Gouldner, 1960), including intimate relationships (Weitkamp et al., 2021). Moreover, because our assessments were cross-sectional and relied on perceived support, it remains difficult to disentangle the directionality of these dynamics. More satisfied partners may indeed provide more support, but they may also simply perceive support as higher because they are embedded in a mutually satisfying relationship. Longitudinal studies with more objective assessments of provided social support are needed to decide which of these alternatives may be closer to reality (Kohler et al., 2024).

However, in close relationships, one partner may either over-benefit or under-benefit from a support exchange. According to the abovementioned equity theory, both scenarios could be equally distressing for both partners. Conversely, social exchange theory suggests that individuals may be more motivated to over-benefit from social exchanges due to self-interest and a tendency to minimize losses in such exchanges (Homans, 1958). This may explain the aforementioned weaker partner effect of received emotional support. Existing studies on social support imbalances in romantic couples have provided mixed findings in light of these classic theories.

On the one hand, under-benefiting is usually associated with poorer relationship well-being than over-benefiting in couples (Grote & Clark, 2001; Sprecher, 2001), which aligns with equity theory. However, Keyes (2002) found that only over-benefiting, not under-benefiting, was associated with poorer emotional well-being. More recently, Wang and Gruenewald (2019) found that under-benefiting is more strongly associated with poorer well-being than over-benefiting, although the magnitude of this association appears to vary across relationship domains, such as family, friendship, and spousal relationships. More specifically, individuals in familial or marital relationships may tolerate dissatisfaction stemming from support imbalances for longer periods than those in friendships, which are generally easier to dissolve. Thus, the consequences of imbalance may also depend on whether dyads are conceptualized as distinguishable or indistinguishable.

Research on intimate relationships in the context of HIV has historically relied on distinguishable dyads, most often differentiating partners by HIV serostatus or gender in heterosexual couples. In contrast, research involving same-gender couples, particularly gay male couples disproportionately affected by HIV, has increasingly adopted indistinguishable dyadic models. In our study, we tested HIV status and sexual orientation composition as potential moderators of social support exchange rather than assuming partner distinguishability a priori. First, when treated appropriately, HIV is now a chronic, medically manageable condition for individuals engaged in effective antiretroviral therapy. Although they are rarely compared directly, existing findings suggest that many serodiscordant and seroconcordant couples function similarly in terms of daily functioning, relationship quality, or psychological adjustment (Faraji et al., 2021; Hou et al., 2025; Remien et al., 2003).

Second, empirical research consistently shows that partners’ sexual orientation is not a central determinant of relationship quality. Large-scale comparative studies have demonstrated that same-gender and heterosexual couples report similar levels of satisfaction, commitment, and relational functioning when assessed under comparable conditions (Gottman et al., 2003; Rostosky & Riggle, 2017). Together, these findings justify modeling HIV-affected couples without presuming inherent partner differences and support a dyadic approach that centers on relational processes rather than categorical distinctions between partners.

The results of our study were consistent with this perspective, as neither HIV status nor the sexual orientation composition of the dyads moderated the actor and partner effects described earlier. Although this counterintuitive finding may partly reflect the limited number of couples in our sample, it may also indicate that key relational processes operate similarly across HIV status and couple composition. Such a null result is nonetheless informative, as it highlights the need for additional research on relational dynamics among HIV-affected couples—particularly those receiving effective medical treatment—that extends beyond a stigma-based framework, which remains relatively rare in the literature (Hou et al., 2025; Martins et al., 2021).

### Limitations

This study has several strengths that may stimulate further advancement in the analysis of the functioning of couples living with HIV, which remains an underdeveloped area in the HIV/AIDS literature. Nevertheless, several limitations should be acknowledged. First, a cross-sectional design precludes causal inferences. More longitudinal studies are needed to better capture couple-level phenomena in the context of HIV/AIDS, including the use of alternative approaches to dyadic data analysis (e.g., the common fate model; Hou et al., 2025). Second, because the dyads were predominantly male and mostly of the same gender, comprising 73.3 % of the sample, the study may have been underpowered to detect between-dyad differences. However, given the well-documented challenges of recruiting HIV-affected couples—particularly in dyadic designs that require disclosure, coordinated participation, and stability of partnership—our sample reflects common constraints in this field (McNutt et al., 2009; Tecimer et al., 2011). As such, these findings may still serve as a valuable stimulus for further research with the potential to deepen the understanding of both universal and condition-specific support processes and to contribute to destigmatizing the experience of living with HIV (Siegel et al., 2018). Third, the participants were highly functioning individuals receiving regular and free medical treatment; therefore, the results may not be generalizable to other populations of PLWH, especially those experiencing socioeconomic adversity or barriers to care (Dincer et al., 2024; Pellowski et al., 2013). Finally, as is typical in studies of social support, support was assessed solely by self-reporting. Nonetheless, subjective perceptions of support are likely central to relationship quality and the long-term functioning of couples (Haber et al., 2007; Melrose et al., 2015).

### Conclusions

A satisfying intimate partnership is a central component of well-being across cultural contexts. In the context of HIV/AIDS, an accumulating body of research has demonstrated that relationship status is an important correlate of psychological well-being and health-related quality of life. The present findings suggest that emotional support exchanged within couples, even when assessed subjectively, functions as a dyadic resource, whereas received emotional support shows a stronger association with each individual’s relationship satisfaction. This pattern did not vary as a function of the partners’ serostatus or the couple’s sexual orientation, indicating that within-dyad processes may be shaped more by the partners’ individual personality characteristics.

The present findings may help inform couple-based counseling for PLWH (Siegel et al., 2018). Interventions should focus on strengthening partners’ capacities to exchange emotional social support within the relationship, regardless of partners’ HIV status, while recognizing that both directions of this exchange are necessary to maintain relationship satisfaction at both the couple and individual levels (Hou & Falconier, 2025). Accordingly, couples may be encouraged to engage in daily behaviors that promote the provision of emotional support, such as active listening and expressions of love and affection. They may also practice receiving support to sustain individual satisfaction by expressing gratitude toward the support provider and communicating their needs. Such activities may foster positive interpersonal exchanges within the couple and contribute to long-term relationship satisfaction or—when necessary—support the development of personal resources for making more constructive interpersonal choices in the future.

## Information about data sharing

All the data are available upon the request from the corresponding author.

## Source of funding

This study was financed by the National Science Center in Poland (research project no 2023/51/B/HS6/00,332).

## Research involving human participants

The study protocol was accepted by the institutional ethics committee.

## Declaration of competing interest

We have nothing to declare.
